# Assessment of Programs Aimed to Decrease or Prevent Mistreatment of Medical Trainees

**DOI:** 10.1001/jamanetworkopen.2018.0870

**Published:** 2018-07-27

**Authors:** Laura M. Mazer, Sylvia Bereknyei Merrell, Brittany N. Hasty, Christopher Stave, James N. Lau

**Affiliations:** 1 Goodman Surgical Education Center, Department of Surgery, Stanford University School of Medicine, Stanford, California; 2 Goodman Surgical Education Center, Stanford–Surgery Policy Improvement Research & Education Center, Department of Surgery, Stanford University School of Medicine, Stanford, California; 3 Lane Medical Library, Stanford University School of Medicine, Stanford, California

## Abstract

**Importance:**

Mistreatment of medical students is pervasive and has negative effects on performance, well-being, and patient care.

**Objective:**

To document the published programmatic and curricular attempts to decrease the incidence of mistreatment.

**Data Sources:**

PubMed, Scopus, ERIC, the Cochrane Library, PsycINFO, and *MedEdPORTAL* were searched. Comprehensive searches were run on “mistreatment” and “abuse of medical trainees” on all peer-reviewed publications until November 1, 2017.

**Study Selection:**

Citations were reviewed for descriptions of programs to decrease the incidence of mistreatment in a medical school or hospital with program evaluation data. A mistreatment program was defined as an educational effort to reduce the abuse, mistreatment, harassment, or discrimination of trainees. Studies of the incidence of mistreatment without description of a program, references to a mistreatment program without outcome data, or a program that has never been implemented were excluded.

**Data Extraction and Synthesis:**

Authors independently reviewed all retrieved citations. Articles that any author found to meet inclusion criteria were included in a full-text review. The data extraction form was developed based on the guidelines for Best Evidence in Medical Education. An assessment of the study quality was conducted using a conceptual framework of 6 elements essential to the reporting of experimental studies in medical education.

**Main Outcomes and Measures:**

A descriptive review of the interventions and outcomes is presented along with an analysis of the methodological quality of the studies. A separate review of the *MedEdPORTAL* mistreatment curricula was conducted.

**Results:**

Of 3347 citations identified, 10 studies met inclusion criteria. Of the programs included in the 10 studies, all were implemented in academic medical centers. Seven programs were in the United States, 1 in Canada, 1 in the United Kingdom, and 1 in Australia. The most common format was a combination of lectures, workshops, and seminars over a variable time period. Overall, quality of included studies was low and only 1 study included a conceptual framework. Outcomes were most often limited to participant survey data. The program outcome evaluations consisted primarily of surveys and reports of mistreatment. All of the included studies evaluated participant satisfaction, which was mostly qualitative. Seven studies also included the frequency of mistreatment reports; either surveys to assess perception of the frequency of mistreatment or the frequency of reports via official reporting channels. Five mistreatment program curricula from *MedEdPORTAL* were also identified; of these, only 2 presented outcome data.

**Conclusions and Relevance:**

There are very few published programs attempting to address mistreatment of medical trainees. This review identifies a gap in the literature and provides advice for reporting on mistreatment programs.

## Key Points

**Question:**

What programmatic and curricular attempts have been reported to decrease the incidence of mistreatment of medical trainees?

**Findings:**

After a systematic review of more than 3300 articles, only 10 peer-reviewed studies presented outcomes from an implemented program to prevent mistreatment. Overall, quality of included studies was low, and few studies reported any outcome data.

**Meaning:**

There are very few published descriptions of programs attempting to decrease mistreatment of medical trainees, and there is a need for improved quantity and quality of such reports.

## Teaser

This review of 10 peer-reviewed studies and 5 program curricula evaluates published programs designed to prevent or decrease the mistreatment of medical trainees.

## Introduction

Mistreatment and abuse of medical trainees has been extensively documented since the 1980s, when Henry Silver drew comparisons between medical students and battered foster children.^1^ In 2014, a systematic review in *Academic Medicine* found 59 studies examining the prevalence of harassment and discrimination in medical schools.^2^ In surveys, questionnaires, interviews, and focus groups, medical trainees at all levels from around the world describe a culture of prevalent and persistent mistreatment.^3,4,5,6^ On the 2014 Association of American Medical Colleges Graduation Questionnaire, excluding reports of public embarrassment, 40% of graduating medical students reported personally experiencing mistreatment.^7^

Mistreatment and abuse of trainees is not just prevalent, it is harmful. Mistreatment is associated with increased burnout,^8^ decreased confidence in clinical abilities,^9^ and symptoms of posttraumatic stress disorder.^10^ And trainees are not the only ones affected: there is mounting evidence that abuse and mistreatment within the care team leads to worse outcomes for patients. A recent Israeli study by Riskin et al^11^ assessed neonatal intensive care unit team performance in a simulation involving a critically ill preterm infant. The participants were informed that an expert would observe the simulation, and the teams were randomly assigned to an expert described as either rude or neutral. Diagnostic and procedural performances were significantly lower in teams exposed to rudeness. The study by Riskin and colleagues provides empirical evidence for the theory that interpersonal aggression can cause iatrogenic events by negatively affecting professionals’ cognitive processing and communication.^12^ In nonmedical workplaces, interpersonal aggression is a well-documented threat to productivity.^13,14^

Mistreatment is pervasive, and its ill effects are felt by perpetrators, targets, and patients. The problem has been documented in multi-institutional,^15^ multispecialty,^16^ and multinational studies^6^ and examined in systematic reviews and meta-analyses.^2^ The incidence of mistreatment has been extensively and repeatedly documented since the 1980s, reflecting the increasing cultural focus worldwide on the topic of harassment in the workplace. Yet there are comparably few articles and no systematic reviews describing attempts to prevent or address mistreatment when it occurs in hospitals or medical schools. The purpose of the current study is to examine institutional efforts to decrease or prevent mistreatment of medical trainees as described in the medical literature or in *MedEdPORTAL*.

## Methods

### Search Strategy

The following digital resources were selected for this systematic review: (1) PubMed (includes MEDLINE); (2) Scopus (a large, multidisciplinary academic database containing most, if not all, of the Embase database); (3) ERIC (an education literature database); (4) the Cochrane Library (contains multiple Cochrane databases, including the Cochrane Database of Systematic Reviews, Database of Abstracts of Reviews of Effects, and the Cochrane Central Register of Controlled Trials); (5) PsycInfo (a psychology literature database); and (6) the Association of American Medical Colleges’ *MedEdPORTAL*. The search terms used for each resource were organized around 2 main themes: students and physician trainees (ie, medical students, interns, residents, and clinical fellows) and mistreatment. Additional search strings were formulated to exclude topics, eg, nursing home residents and elder abuse (vs abuse of physician trainees). Searches were crafted to take advantage of databases’ controlled vocabularies (subject headings) when available. These headings were combined with additional searches targeting titles, author keywords, and abstracts. All searches were limited to English. Editorials and letters to the editor were excluded. The systematic review sections of the Preferred Reporting Items for Systematic Reviews and Meta-analyses (PRISMA) reporting guideline were followed. Search terms and the entire search strategy for each database are available in the eAppendix in the Supplement. The search strategy was created cooperatively by the authors, including a medical librarian (C.S.), a PhD educator (S.B.M.), and clinicians with experience creating and evaluating mistreatment programs at the medical school and residency level (L.M.M., B.N.H., and J.N.L.). Because this article reviewed previously published studies and did not involve human participants, it was not submitted to an institutional review board.

### Inclusion and Exclusion Criteria

We included articles that described an implemented mistreatment program in a medical school or hospital setting. We defined a mistreatment program as an educational effort designed to reduce the abuse, mistreatment, or harassment of trainees or discrimination against trainees. We excluded reports of the incidence of mistreatment without description of a program, references to a mistreatment program with no details of the intervention or with no outcome data, or a program that had never been implemented. We also excluded non-English studies and studies published without peer review.

All articles from the initial search strategy were imported into the Covidence systematic review management software platform. Three of us (L.M.M., S.B.M., and B.N.H.) independently reviewed the titles and abstracts of all articles retrieved with the search strategy described. All articles felt to meet inclusion criteria by any author were included in the full-text review. We (L.M.M., S.B.M., and B.N.H.) then completed the full-text review independently. Disagreements were discussed until full consensus was reached and the final list of included studies was selected for full data extraction and analysis.

### Data Extraction and Analysis

The data extraction form was jointly developed by all authors based on the guidelines for Best Evidence in Medical Education (BEME).^17^ The included studies were reviewed and entered into the data extraction form. In addition to bibliographic data and details of the included interventions, we performed a basic assessment of the study quality using the 6 elements identified by Cook et al^18^ as essential to quality reporting of experimental studies in medical education. Specifically, the quality assessment elements were (1) a thorough review of the literature, (2) a statement or implication of a conceptual framework as programmatic guiding principle, (3) a clear statement of the mistreatment program’s intent or the research study intent, (4) a study design description, including type and additional features, (5) a description of the study intervention and control group if applicable, and (6) a statement of human subjects research.

We distinguished between published articles, found in the first 5 listed databases (PubMed, Scopus, ERIC, Cochrane Library, and PsycInfo), and educational resources, published in *MedEdPORTAL*. Because of differences in the format and focus of *MedEdPORTAL* vs the other search engines, we elected to evaluate the 5 curricula that went to the data extraction stage from *MedEdPORTAL* separately from the articles found in the other databases. In particular, we did not assess these resources using the framework from Cook et al for study quality because they were presented as curricular resources, not research.

## Results

The initial search produced 3347 citations and included all peer-reviewed publications through November 2017. This was reduced to 38 potentially relevant articles that we included in the full-text review. Twenty-eight were excluded, most because they did not include a description of the mistreatment program (Figure). Ten studies were included in the final review.^19,20,21,22,23,24,25,26,27,28^ Of note, this flowchart does not include the results from the *MedEdPORTAL* search, which are reported separately.^25,29,30,31,32^

**Figure.**
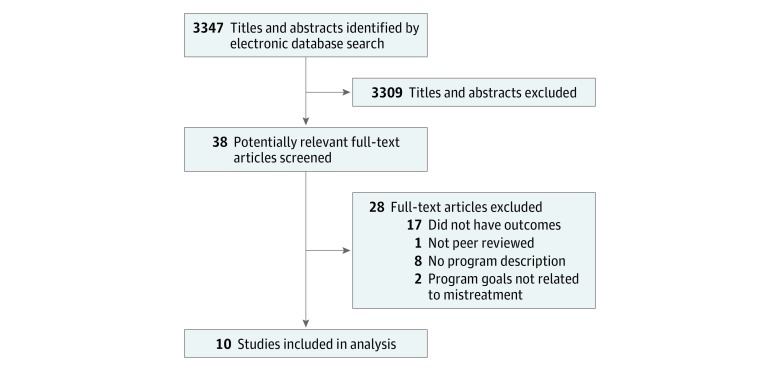
Selection Strategy for the Full Literature Review of Published Mistreatment Programs

We report results in 3 major sections. Initially we present a descriptive review of the included studies, their mistreatment programs and definitions, and outcomes (Table 1 and Table 2). Next, we present a discussion of the methodological quality of these studies based on the framework developed by Cook et al^18^ for evaluating the quality of medical education reporting (Table 3). Finally, we present a separate review of the mistreatment curricula available on *MedEdPORTAL*, as these reports do not necessarily follow the guidelines for medical education research, focusing instead on the principles of curriculum development and evaluation (Table 4).

**Table 1.** Summary of Included Studies of Mistreatment Programs

Source	Institution	Participants^a^	Beneficiaries^a^	Program	Outcomes
Moscarello et al,^19^ 1996	University of Toronto	Faculty (No. not reported) and medical students (n = 327)	Medical students	Seminars and lectures	Surveys showed no change in rates of noncontact sexual harassment and a decrease in contact sexual harassment
Jacobs et al,^20^ 2000	Stanford University	Faculty (n = 50 for initial pilot; then compulsory for all faculty)	Faculty and students	1- to 2-d retreats	Decreased perception of hostile work environment
Heru,^21^ 2003	Brown Medical School	Psychiatry residents (n = 14)	Psychiatry residents	Participation as actors in videos depicting mistreatment	Positive learner perceptions on open-ended surveys
Fried et al,^22^ 2012	University of California, Los Angeles	Faculty, residents, medical students, nurses (No. not reported)	Medical students	13-y multipronged program including workshops and increased reporting options	No change in frequency, severity, or type of mistreatment reports
Cresswell et al,^23^ 2015	North Middlesex University Hospital	Residents (total No. not reported; mean 20 residents per workshop)	Residents	Half-day workshop	High learner satisfaction with the program, improved participant attitudes toward mistreatment
Wagner et al,^24^ 2015	University of California, Los Angeles	Medical students on surgery clerkship (n = 187)	Medical students on surgery clerkship	Anonymous web-based reporting mechanism	High student satisfaction, increased interest in surgery as a career, no change in number of mistreatment reports
Fleit et al,^25^ 2017	Stony Brook University School of Medicine	Medical students, residents, faculty, leadership, clinical staff members (No. not reported)	Medical students	Multipronged, including statement of faculty behavioral expectations, increased reporting options, and small group discussions	Trend toward decreased reports of mistreatment, increased percentage of students never having experienced mistreatment
Lau et al,^26^ 2017	Stanford University	Medical students on surgery clerkship (n = 164)	Medical students on surgery clerkship	8-wk seminar series with video triggers and group discussion	Positive student evaluations, reduced reports of mistreatment
Scott et al,^27^ 2017	University of Sydney	Medical students (n = 30)	Medical students	Three 3-h drama-based workshops	Positive student evaluations
Smith-Coggins et al,^28^ 2017	Stanford University	Medical students, residents, faculty (No. not reported)	Medical students	Institution-wide, including increased reporting options, tool kits with strategies to prevent mistreatment, and small group discussions	Increased student awareness of policy, reduced reports of mistreatment

^a^ *Participants* refers to the groups included in the intervention. *Beneficiaries* refers to the group(s) the program intended to assist. For example, an intervention may have required faculty to attend workshops (participants) to decrease mistreatment of medical students (beneficiaries).

**Table 2.** Description and Definition of Mistreatment Types of Included Studies of Mistreatment Programs

Source	Types of Mistreatment Included
Moscarello et al,^19^ 1996	Sexual harassment: divided into noncontact (including being stared at or ogled; unwelcome remarks, jokes, or innuendos; or being shown pornographic images) and contact (physical contact including touching, pinching, or sexual intimacy)
Jacobs et al,^20^ 2000	Sexual harassment: repeated and unwanted sexual behavior or creation of a hostile environment
	Gender insensitivity: comments or actions that intentionally or unintentionally devalue the individual because of his or her sex
Heru,^21^ 2003	Not defined
Fried et al,^22^ 2012	Physical: slapped, struck, or pushed
	Verbal: yelled or shouted at, called a derogatory name, cursed, or ridiculed
	Sexual: inappropriate physical or verbal advances, intentional neglect, or sexual jokes
	Ethnic: intentional neglect, ethnic jokes, comments or expectations regarding stereotypical behavior
	Power: made to feel intimidated, dehumanized, or threatened based on grades, recommendations, or career
Cresswell et al,^23^ 2015	Bullying: words, actions, or conduct that ridicules, intimidates, or threatens and affects individual dignity and well-being; “It is largely identified not so much by what has actually been done, but rather by the effect that it has on the individual.”
Wagner et al,^24^ 2015	Not defined
Fleit et al,^25^ 2017	“Physical, verbal or emotional behavior that shows disrespect for medical students and unreasonably interferes with their learning process”
Lau et al,^26^ 2017	“The definition of mistreatment is subjective and heavily context based”; specific examples include public humiliation
Scott et al,^27^ 2017	Not defined
Smith-Coggins et al,^28^ 2017	“Exploitation, harassment, and humiliation”

**Table 3.** Quality Assessment of Included Studies of Mistreatment Programs

Source	Literature Review^a^	Conceptual Framework^b^	Study Intent^c^	Study Design	Definition of Intervention and Control Groups	IRB Human Subjects Approval
Moscarello et al,^19^ 1996	Minimal	None	Not stated	Single group, pretest and posttest	Historical control with no intervention	Not stated
Jacobs et al,^20^ 2000	Minimal	None	Stated	Single group, pretest and posttest	Single group surveyed twice during a longitudinal series of interventions	Not stated
Heru,^21^ 2003	Moderate	None	Not stated	Single group, posttest only	No control group	Not stated
Fried et al,^22^ 2012	Moderate	None	Stated	Cross-sectional	No control group	Stated
Cresswell et al,^23^ 2015	Minimal	None	Stated	Single group, posttest only	No control group	Not stated
Wagner et al,^24^ 2015	Moderate	None	Stated	Single group, pretest and posttest	Historical control with no intervention	Stated
Fleit et al,^25^ 2017	Significant	None	Stated	Cross-sectional	Historical control	Stated
Lau et al,^26^ 2017	Moderate	None	Stated	Single-group, pretest and posttest	Historical control	Stated
Scott et al,^27^ 2017	Minimal	Professional identity formation	Stated	Single-group, pretest and posttest	No control group	Stated
Smith-Coggins et al,^28^ 2017	Minimal	None	Stated	Cross-sectional	Historical control	Stated

Abbreviation: IRB, institutional review board.

^a^ *Minimal* refers to articles that cited studies to establish the prevalence of mistreatment or its impact; *moderate* refers to any attempts to synthesize or draw conclusions from the cited articles; *significant* refers to critical discussion of the literature, including an assessment of its quality and notable gaps.

^b^ *Conceptual framework* refers not to a theory on why mistreatment is harmful, but why the proposed intervention was created or an explanation for the intervention’s success or failure.

^c^ *Study intent* refers to either the purpose of the program or the research study.

**Table 4.** Summary of *MedEdPORTAL* Curricula of Implemented Mistreatment Programs

Source	Institution	Participants	Beneficiaries	Program	Outcomes
Reddy et al,^29^ 2013	University of Chicago Pritzker School of Medicine	Medical students	Medical students	Interactive workshop with PowerPoint slides	None
Rich et al,^30^ 2015^a^	University of Vermont	Medical students, residents, faculty	Medical students	Video-based discussion session	None
Lewis et al,^32^ 2015^a^	University of Vermont	Medical students, residents, faculty	Medical students	Web module for students to complete at home	None
Mazer et al,^31^ 2015	Stanford University	Medical students on surgery clerkship	Medical students on surgery clerkship	Two video-based discussion sessions with clerkship director	Positive learner satisfaction
Fleit et al,^25^ 2017	Stony Brook University	Medical professionals and trainees	Medical students and trainees	Six case-study videos followed by discussion session	Positive learner satisfaction, increased awareness of policies

^a^ These 2 curricula present the same material in a workshop vs electronic module format.

### Descriptive Analysis of Included Studies

#### Setting

All 10 included studies were set at an academic medical center. Seven programs were in the United States, 1 in Canada, 1 in the United Kingdom, and 1 in Australia.

#### Program Descriptions

While similar in aims, the included mistreatment programs were diverse in conception. The most common format was a combination of lectures, workshops, and seminars occurring over a variable time period. Eight of the included studies described some combination of lectures and workshops.^19,20,22,23,25,26,27^ They ranged from a half-day workshop^23^ to a 13-year experience with a wide variety of programs.^22^ Two studies used video scenarios that depicted mistreatment events.^21,26^ Many of the programs were multifaceted, and, in addition to lectures or workshops, included increased mechanisms for reporting mistreatment.^22,24,25,28^ One program comprised exclusively a novel reporting mechanism allowing for anonymous and web-based reporting of mistreatment events during a surgical clerkship, with real-time responses from the clerkship director.^24^ Of 10 programs, 4 (40%) were voluntary, 2 (20%) were compulsory, 2 (20%) included both voluntary and compulsory components, and 2 (20%) did not specify. Descriptive summaries of mistreatment programs appear in Table 1.

The size of the intervention and the number of participants varied among programs. Heru^21^ described focus groups of 14 residents making videotapes to depict mistreatment. At the opposite end of the spectrum, Fried et al^22^ created a longitudinal 13-year program with multiple facets, affecting more than 2000 students and an unknown number of residents and faculty.

#### Motivation, Program Goals, and Beneficiaries

Eight of the 10 studies had explicit statements of the program’s goals. The stated goals were to either increase awareness of mistreatment or reduce its incidence. Some studies specifically targeted 1 type or definition of mistreatment; these are described in Table 2. We distinguished between the program participants, who attended the workshops, lectures, or other intervention activities, and the program beneficiaries, or that group that the program identified as the target of mistreatment. Seven programs aimed to benefit medical students, 2 aimed to benefit residents, and 1 aimed to benefit both faculty and students. Most programs were designed for the beneficiary group to attend and participate. In 3 programs designed to reduce mistreatment of medical students, faculty and other health care personnel also participated in the programmatic activities.

#### Outcomes

The program outcome evaluations consisted primarily of surveys and reports of mistreatment. All of the included studies evaluated participant satisfaction, which was mostly qualitative. Seven studies also included the frequency of mistreatment reports; either surveys to assess perception of the frequency of mistreatment or the frequency of reports via official reporting channels. Wagner et al^24^ situated their programs within a surgical clerkship and also assessed interest in surgery as a career and student satisfaction with the clerkship as a whole. Overall, participant satisfaction was high in all studies. For studies reporting preintervention and postintervention mistreatment report data, there were minimal changes overall. One program designed to address sexual harassment reported no change in rates of noncontact harassment and a decrease in incidences of contact sexual harassment.^19^ In the longest-running program, described by Fried et al,^22^ a 13-year program resulted in no change in the frequency or type of mistreatment reports. Outcome data are reported in Table 2.

#### Definitions of Mistreatment

As mistreatment is a broad concept, we also examined what, if any, definition was provided by the study for the concept of mistreatment (Table 2). Seven studies provided specific definitions of mistreatment. Two studies^19,20^ focused specifically on sexual mistreatment, one^23^ defined bullying, and one^22^ provided definitions for a variety of categories of mistreatment, including sexual, physical, and verbal. Two studies^21,24^ did not define mistreatment in their description.

#### Costs and Resources Needed

The costs required for each program also vary significantly. The most basic program described is a web-based reporting system that would be essentially free to run and require no additional personnel, training, or resources.^24^ No program specifically listed costs or resources. A consistent theme, however, was personalized attention from individuals in a position of authority. For programs that existed at a more local level, in 1 clerkship, direct oversight by the clerkship or program director was a consistent factor and may contribute to programmatic success.^24,26^ Many of the programs involved creating videos to portray mistreatment, and video production is costly. The *MedEdPORTAL* curricula, however, include the videos, cases, and worksheets and have the potential to significantly decrease future costs to medical schools or hospitals wishing to replicate the program.

### Methodological Quality

Methodological quality is summarized in Table 3. Background literature review was rated minimal if it included citations documenting the prevalence or impact of mistreatment. Moderate literature review included attempts to synthesize the literature or draw conclusions, and significant review refers to critical discussion of the literature with an assessment of its quality and gaps. One study of 10 was thought to present a significant literature review and 4 presented moderate background review. Eight of the included studies had an explicit statement of study intent, which we defined as a statement of either the mistreatment program’s intent or the intent of the research study. Only 1 study had a conceptual framework, which was introduced in the introduction but not directly linked to the study outcomes.^27^ The most common study design was the single-group pretest and posttest (7 of 10 studies), followed by cross-sectional design (3 of 10 studies). Four studies did not document approval or exempt status by the institutional review board.

### Published Curricula in *MedEdPORTAL*

We also evaluated *MedEdPORTAL* publications for peer-reviewed, implemented mistreatment programs that have not been submitted as a research study. The initial search strategy revealed 5 mistreatment programs on *MedEdPORTAL*. All 5 had specific educational aims, including an increased awareness of mistreatment and of institutional resources. None of the curricular descriptions provided the number of program participants. Two of the programs provided outcome data in the form of open-ended student satisfaction surveys and represented the curricular components of 2 of the research reports already described in this article.^25,31^ The *MedEdPORTAL* curricula are summarized in Table 4.

## Discussion

The prevalence of mistreatment has been repeatedly documented in the literature, and there is mounting evidence that abuse of medical students and residents harms both clinicians and patients. Given the pervasiveness of the problem across nationalities and specialties, we hypothesized that many academic medical centers may be moving beyond diagnosis and toward programs intended to prevent mistreatment within the medical team. After a review of more than 3300 articles, we found only 10 peer-reviewed studies presenting outcomes from an implemented program to decrease the impact of mistreatment. A review of *MedEdPORTAL* revealed an additional 5 curricula (2 within 1 program) on mistreatment, for a total of 15 peer-reviewed, published mistreatment programs. In comparison, a 2014 review article found 59 studies describing the prevalence of mistreatment.

In a review of study quality for the 10 research studies, we found that background literature review was mostly minimal to moderate. Two articles had no explicit statement of intent, either for the mistreatment program or for the research study. Only 1 of the included articles had an explicitly stated conceptual framework, and most outcomes were learner satisfaction posttest–only designs. Almost half the studies did not cite institutional review board approval. Of the 5 curricula published on *MedEdPORTAL*, none included a conceptual framework and only 2 presented outcome data. The programs are very diverse in concept, content, and outcome measures, preventing any real conclusions regarding best practices for future educators wishing to address this problem.

### Limitations

There are potential limitations to our review. Although all attempts were made to complete a thorough search of the identified databases, the sheer diversity of terms for *mistreatment* may have led to omissions in our search. There is also a significant possibility that publication bias may exist in the reporting of mistreatment programs with negative results. Additionally, we looked exclusively at reports of programs to prevent mistreatment of trainees. Abuse and harassment in the workplace can also affect professionals, especially in a hospital setting where divergent power dynamics, such as those between physicians and nurses, can mimic the student-teacher relationship. This may be a valuable target for future studies.

Despite these limitations, several implications emerge from this review that can help educators seeking to affect the culture of mistreatment and create change within their institutions.

### Identification of Conceptual Frameworks

Mistreatment is a complex and often misunderstood concept. While it is tempting to say of mistreatment that we will “know it when we see it,” the evidence is that different groups may in fact interpret mistreatment differently and account for mitigating or contextual factors.^33,34^ The included articles in this review cover a broad range of actions, and it is not clear that all of these mistreatment behaviors share the same root cause or have their solution in the same program. The introduction and background sections of the included articles are devoted almost entirely to proving that mistreatment is a problem, rather than developing a theoretical framework linking a cause with a proposed solution. The article by Jacobs et al^20^ argues that sexism in any form causes long-lasting impact on institutional equanimity and productivity. The work of Heru^21^ equates mistreatment with a lack of professionalism. While understanding the impact of mistreatment on individuals, the system, and the profession is important, attempts to fix the problem require theories that explain why a proposed program will work. Clarification studies, asking how and why a program works, are essential to deepen our understanding of the problem and pave the way toward lasting, effective solutions.^35,36^ We suggest that educators seeking to implement or create a mistreatment program begin with a clear definition of the problem and a critical evaluation of the literature, including social science literature, to develop a theoretical framework to guide program development. A theoretical framework can also help identify appropriate outcome measures to gauge the success of any mistreatment program, as relevant outcomes can include not just occurrences of mistreatment but also recognition of policies and reporting measures, as well as evidence of broader change in the institutional culture.

### Publication of Curriculum Descriptions

We included *MedEdPORTAL* as a database in our initial search, something we have not observed in other review articles on medical curricula. We found 5 publications from 4 institutions, a significant number in this relatively small field, although 2 of these publications provided curricular materials for programs whose outcomes were described in the research articles. Given the prevalence of the problem, it is unlikely that only 13 programs have attempted to address medical student or resident mistreatment. It is possible that programs with negative results are not being published, or that implemented programs have minimal outcome data. We would strongly recommend both (1) strategic planning to research and/or evaluate the implementation process and impact of mistreatment programs and (2) publishing of implemented curricula in peer-reviewed form, as either research articles or curricular products. We recommend that future publications include as much detail as possible to allow other institutions to replicate successful efforts; specifically, details regarding cost or time resources are especially important. Increased publication of the impact of mistreatment programs will answer the question of whether attempts to solve the problem of mistreatment are as widespread as the attempts to diagnose it (we compare our 10 included studies describing mistreatment programs with the 59 studies included in a recent review article documenting the prevalence of mistreatment).^2^ The publication of curricula will also allow educators to learn from one another and avoid recreating existing materials. Additionally, all of the included studies involved a single institution and most a single department. Sharing curricula online may pave the way for multi-institutional collaborations. Finally, the current review emphasizes the potential importance of including *MedEdPORTAL* as an electronic database for review articles in medical education.

### Multiple Sources of Outcome Data

It is extremely challenging to evaluate mistreatment programs because outcomes may be collected from students, residents, faculty, end-of-rotation evaluations, and other sources. One potential risk is that raising awareness of mistreatment may cause a subsequent increase in reports of mistreatment. This (hopefully temporary) increase most likely results from increased understanding of the problem and improved reporting mechanisms rather than a true increase in mistreatment. This may be the case in 2 of the included studies, where increased reporting options for mistreatment and increased institutional efforts to respond to the reports did nothing to decrease reporting frequency.^22,24^ Numbers of mistreatment reports are not sufficient criteria, and learner satisfaction, attitudes, and perceptions are essential additional components of mistreatment program evaluation.

## Conclusions

Mistreatment is pervasive and harmful but often poorly defined. We found 10 research studies and 5 curricular descriptions representing a total of 13 programs to decrease the incidence of mistreatment in academic medical centers. We emphasize the need to continue addressing this problem, not just with acknowledgment of its existence but with proactive programs to change the academic medical culture. We stress the need for a theoretical framework for mistreatment programs to ensure that educators, participants, and beneficiaries have a shared understanding of the problem and can therefore evaluate the success of any proposed solutions. This review identifies a clear need for additional and more extensive studies on this topic.

## Notes

Supplement. **eAppendix.** Search Strategy

## References

[1] Silver HK Medical students and medical school. JAMA. 1982;247(3):-. doi:10.1001/jama.1982.03320280029024 7054531

[2] Fnais N, Soobiah C, Chen MH, Harassment and discrimination in medical training: a systematic review and meta-analysis. Acad Med. 2014;89(5):817-827. doi:10.1097/ACM.0000000000000200 24667512

[3] Nagata-Kobayashi S, Sekimoto M, Koyama H, Medical student abuse during clinical clerkships in Japan. J Gen Intern Med. 2006;21(3):212-218. doi:10.1111/j.1525-1497.2006.00320.x 16390504 PMC1828085

[4] Rees CE, Monrouxe LV “A morning since eight of just pure grill”: a multischool qualitative study of student abuse. Acad Med. 2011;86(11):1374-1382. doi:10.1097/ACM.0b013e3182303c4c 21952053

[5] Shoukat S, Anis M, Kella DK, Prevalence of mistreatment or belittlement among medical students—a cross sectional survey at a private medical school in Karachi, Pakistan. PLoS One. 2010;5(10):e13429. doi:10.1371/journal.pone.0013429 20976173 PMC2955546

[6] Uhari M, Kokkonen J, Nuutinen M, Medical student abuse: an international phenomenon. JAMA. 1994;271(13):1049-1051. doi:10.1001/jama.271.13.1049 8139064

[7] Association of American Medical Colleges Medical School Graduation Questionnaire: 2014 All Schools Summary Report. Washington, DC: Association of American Medical Colleges; 2015. https://www.aamc.org/download/464412/data/2016gqallschoolssummaryreport.pdf. Accessed November 1, 2017.

[8] Cook AF, Arora VM, Rasinski KA, Curlin FA, Yoon JD The prevalence of medical student mistreatment and its association with burnout. Acad Med. 2014;89(5):749-754. doi:10.1097/ACM.0000000000000204 24667503 PMC4401419

[9] Schuchert MK The relationship between verbal abuse of medical students and their confidence in their clinical abilities. Acad Med. 1998;73(8):907-909. doi:10.1097/00001888-199808000-00018 9736853

[10] Heru A, Gagne G, Strong D Medical student mistreatment results in symptoms of posttraumatic stress. Acad Psychiatry. 2009;33(4):302-306. doi:10.1176/appi.ap.33.4.302 19690110

[11] Riskin A, Erez A, Foulk TA, The impact of rudeness on medical team performance: a randomized trial. Pediatrics. 2015;136(3):487-495. doi:10.1542/peds.2015-1385 26260718

[12] Suresh G, Horbar JD, Plsek P, Voluntary anonymous reporting of medical errors for neonatal intensive care. Pediatrics. 2004;113(6):1609-1618. doi:10.1542/peds.113.6.1609 15173481

[13] Estes B, Wang J Integrative literature review: workplace incivility: impacts on individual and organizational performance. Hum Resource Dev Rev. 2008;7(2):218-240. doi:10.1177/1534484308315565

[14] Pearson CM, Porath CL On the nature, consequences and remedies of workplace incivility: no time for “nice”? think again. Acad Manage Perspect. 2005;19(1):7-18. doi:10.5465/ame.2005.15841946

[15] Baldwin DC Jr, Daugherty SR, Eckenfels EJ Student perceptions of mistreatment and harassment during medical school: a survey of ten United States schools. West J Med. 1991;155(2):140-145. 1926843 PMC1002944

[16] Cook DJ, Liutkus JF, Risdon CL, Griffith LE, Guyatt GH, Walter SD Residents’ experiences of abuse, discrimination and sexual harassment during residency training: McMaster University Residency Training Programs. CMAJ. 1996;154(11):1657-1665. 8646653 PMC1487906

[17] Harden RM, Grant J, Buckley G, Hart IR Best evidence medical education. Adv Health Sci Educ Theory Pract. 2000;5(1):71-90. doi:10.1023/A:1009896431203 12386477

[18] Cook DA, Beckman TJ, Bordage G Quality of reporting of experimental studies in medical education: a systematic review. Med Educ. 2007;41(8):737-745. doi:10.1111/j.1365-2923.2007.02777.x 17661881

[19] Moscarello R, Margittai KJ, Rossi MF Impact of faculty education on the incidence of sexual harassment experienced by Canadian medical students. J Womens Health. 1996;5(3):231-237. doi:10.1089/jwh.1996.5.231

[20] Jacobs CD, Bergen MR, Korn D Impact of a program to diminish gender insensitivity and sexual harassment at a medical school. Acad Med. 2000;75(5):464-469. doi:10.1097/00001888-200005000-00017 10824771

[21] Heru AM Using role playing to increase residents’ awareness of medical student mistreatment. Acad Med. 2003;78(1):35-38. doi:10.1097/00001888-200301000-00008 12525407

[22] Fried JM, Vermillion M, Parker NH, Uijtdehaage S Eradicating medical student mistreatment: a longitudinal study of one institution’s efforts. Acad Med. 2012;87(9):1191-1198. doi:10.1097/ACM.0b013e3182625408 22836847 PMC4399975

[23] Cresswell K, Sivashanmugarajan V, Lodhi W, Yoong W Bullying workshops for obstetric trainees: a way forward. Clin Teach. 2015;12(2):83-87. doi:10.1111/tct.12261 25789891

[24] Wagner JP, Tillou A, Nguyen DK, Agopian VG, Hiatt JR, Chen DC A real-time mobile web-based module promotes bidirectional feedback and improves evaluations of the surgery clerkship. Am J Surg. 2015;209(1):101-106. doi:10.1016/j.amjsurg.2014.08.035 25454963

[25] Fleit HB, Iuli RJ, Fischel JE, Lu WH, Chandran L A model of influences on the clinical learning environment: the case for change at one U.S. medical school. BMC Med Educ. 2017;17(1):63. doi:10.1186/s12909-017-0900-9 28335770 PMC5364543

[26] Lau JN, Mazer LM, Liebert CA, Bereknyei Merrell S, Lin DT, Harris I A mixed-methods analysis of a novel mistreatment program for the surgery core clerkship. Acad Med. 2017;92(7):1028-1034. doi:10.1097/ACM.0000000000001575 28121657

[27] Scott KM, Berlec Š, Nash L, Grace under pressure: a drama-based approach to tackling mistreatment of medical students. Med Humanit. 2017;43(1):68-70. doi:10.1136/medhum-2016-011031 28228573

[28] Smith-Coggins R, Prober CG, Wakefield K, Farias R Zero tolerance: implementation and evaluation of the Stanford Medical Student Mistreatment Prevention Program. Acad Psychiatry. 2017;41(2):195-199. doi:10.1007/s40596-016-0523-1 27093963

[29] Reddy S, Ogden P, Arora V, Is it mistreatment? mistreatment education for medical students entering clinical training. *MedEdPORTAL* 2013;9:9569. 10.15766/mep_2374-8265.9569

[30] Rich A, Ackerman S, Patel C, Feldman N, Adams D, Lewis J Creating a positive learning environment: educational film and discussion guide. *MedEdPORTAL* 2015;11:10131. 10.15766/mep_2374-8265.10131

[31] Mazer L, Liebert C, Bereknyei Merrell S, Lin D, Lau J Establishing a positive clinical learning environment in the surgery core clerkship: a video-based mistreatment curriculum. *MedEdPORTAL**.* 2015;11:10313. 10.15766/mep_2374-8265.10313

[32] Lewis J, Fedlman N, Rich A, Ackerman S, Patel C Positive learning environment and mistreatment prevention module. *MedEdPORTAL**.* 2015;11:10113. 10.15766/mep_2374-8265.10113

[33] Brandford E, Hasty B, Bruce JS, Underlying mechanisms of mistreatment in the surgical learning environment: a thematic analysis of medical student perceptions. Am J Surg. 2018;215(2):227-232. doi:10.1016/j.amjsurg.2017.10.042 29167023

[34] Ogden PE, Wu EH, Elnicki MD, Do attending physicians, nurses, residents, and medical students agree on what constitutes medical student abuse? Acad Med. 2005;80(10)(suppl):S80-S83. doi:10.1097/00001888-200510001-00022 16199465

[35] Hasty BN, Miller SE, Bereknyei Merrell S, Lin DT, Shipper ES, Lau JN Medical student perceptions of a mistreatment program during the surgery clerkship. Am J Surg. 2018;215(4):761-766. doi:10.1016/j.amjsurg.2018.01.001 29395030

[36] Cook DA, Bordage G, Schmidt HG Description, justification and clarification: a framework for classifying the purposes of research in medical education. Med Educ. 2008;42(2):128-133. doi:10.1111/j.1365-2923.2007.02974.x 18194162

