# Supplementary material for: Assessment of Programs Aimed to Decrease or Prevent Mistreatment of Medical Trainees
Source: JAMA Netw Open. 2018 Jul 27;1(3):e180870. doi: 10.1001/jamanetworkopen.2018.0870 (PMC6324298; doi:10.1001/jamanetworkopen.2018.0870)
Supplement: Supplement. — eAppendix. Search Strategy [file jamanetwopen-1-e180870-s001.pdf]

Supplementary Online Content

Mazer LM, Bereknyei Merrell S, Hasty BN, Stave C, Lau JN. Assessment of programs aimed to decrease or prevent mistreatment of medical trainees. *JAMA Netw Open*. 2018;1(3):e180870. doi:10.1001/jamanetworkopen.2018.0870

eAppendix. Search Strategy

This supplementary material has been provided by the authors to give readers additional information about their work.

© 2018 Mazer LM et al. *JAMA Network Open.*

eAppendix: Search Strategy

Sample search terms

Trainees	internship, interns, residents, medical students, clerkship, trainees, housestaff, clinical fellows
Mistreatment	mistreatment, cyberbullying, bullying, abuse, maltreatment, harassment, humiliation, yelling, intimidation, racism, coercion, discrimination, disrespect, disruptive behavior, unprofessionalism, hostility, cruelty, assault
Excluded concepts	nursing homes, domestic violence, elder abuse, editorials, letters

PUBMED

("Education, Medical, Graduate" [mesh] OR "Clinical Clerkship" [mesh] OR "Education, Medical, Undergraduate" [mesh] OR interns [ti] OR intern [ti] OR "Internship and Residency" [mesh] OR "medical student" [tw] OR "medical students" [tw] OR "Students, Medical" [mesh] OR clerkship* [tw] OR residents [ti] OR trainee* [ti] OR housestaff [ti] OR "clinical fellow" [tw] OR fellows [tw]) AND (Mistreat* [ti] OR "bullying" [mesh] OR cyberbully* [tw] OR bully* [tw] OR abuse [tw] OR maltreat* [tw] OR abused [tw] OR harass* [tw] OR humiliate* [tw] OR mistreat* [tw] OR yelling [tw] OR "verbal abuse" [tw] OR "physical abuse" [tw] OR intimidate* [tw] OR coercion [tw] OR ((sex [tw] OR gender [tw] OR ethic* [tw] OR ethnic* [tw] OR obes* [tw] OR racial [tw] OR cultural [tw]) AND bias* [tw]) OR "coercion" [mesh] OR "sexual abuse" [tw] OR racism [tw] OR discrimination [tw] OR discriminated [tw] OR "Violence"[Mesh:NoExp] OR "Workplace Violence"[Mesh] OR "Aggression" [mesh] OR "Power (Psychology)" [mesh] OR "Prejudice" [mesh] OR "Verbal Behavior" [mesh] OR "Punishment" [mesh] OR unprofessional* [tw] OR disrespect* [tw] OR "disruptive behavior" [tw] OR cruel* [tw] OR hostil* [tw]) NOT ("elder abuse" [tw] OR "Elder Abuse" [mesh] OR (resident* [ti] AND "nursing home" [ti]) OR "Homes for the Aged" [mesh]) AND English [lang] NOT ("editorial" [pt] OR "letter" [pt]) NOT ("domestic violence" [mesh] OR "domestic violence" [ti])

SCOPUS

· Paste the search into the Advance Search tab
· This is a combination of Title and Keyword (aka, subject heading) searches. I avoided abstracts as they were too non-specific

(((TITLE("internship" OR "intern" OR residents OR residency OR resident OR "medical student*" OR clerkship* OR trainee* OR housestaff OR "clinical fellow" OR fellows) AND KEY(("medical student" OR "medical school" OR "resident")))) AND ((TITLE(mistreat* OR

© 2018 Mazer LM et al. *JAMA Network Open.*

cyberbully* OR bully* OR abuse OR maltreat* OR abused OR harass* OR humiliate* OR yelling OR intimidate* OR coercion OR racism OR discrimination OR discriminated OR disrespect* OR "disruptive behavior" OR unprofessional* OR hostil* OR cruel*)) OR (TITLE((sex OR gender OR ethic* OR ethnic* OR obes* OR racial OR cultural) AND bias*)) OR (KEY(Abuse OR aggression OR "professional misconduct" OR harass* OR assault* OR maltreat* OR racism OR bullying OR hostil*)))) AND NOT ((KEY("nursing home*" OR "Domestic violence")) OR (TITLE("domestic violence" OR (resident* AND "nursing home*") OR "elder abuse"))) AND (LIMIT-TO(LANGUAGE,"English")) AND (EXCLUDE(DOCTYPE,"le") OR EXCLUDE(DOCTYPE,"no")) AND (EXCLUDE(DOCTYPE,"ed"))

· **TITLE**: “internship” OR “intern” OR residents OR residency OR resident OR “medical student*” OR “undergraduate medical student*” OR “graduate medical student*” OR clerkship* OR trainee* OR housestaff OR “clinical fellow” OR fellows OR **KEYWORD**: (“medical student” OR “medical school” OR “resident”)

· **TITLE**: (mistreat* OR cyberbully* OR bully* OR abuse OR maltreat* OR abused OR harass* OR humiliate* OR yelling OR intimidate* OR coercion OR racism OR discrimination OR discriminated OR disrespect* OR "disruptive behavior" OR unprofessional* OR hostil* OR cruel*) OR ((sex OR gender OR ethic* OR obes* OR racial OR cultural) AND bias*) OR **KEYWORD**: Abuse OR aggression OR “professional misconduct” OR harass* OR assault* OR maltreat* OR racism OR bullying OR hostil*

· **TITLE**: “domestic violence” OR (resident* AND “nursing home*”) OR “elder abuse” OR **KEYWORD**: “nursing home*” OR Domestic violence

1 AND 2 AND English NOT 3 NOT letters NOT notes NOT editorials

ERIC (EBSCO Host)

Like the SCOPUS search, focused on Titles and Subject Headings

1. SU ((“internship” OR “intern” OR residents OR residency OR resident OR “medical student” OR “medical students” OR clerkship* OR trainee* OR housestaff OR “clinical fellow” OR fellows)) AND SU ((mistreat* OR cyberbully* OR bully* OR abuse OR maltreat* OR abused OR harass* OR humiliate* OR yelling OR intimidate* OR coercion OR racism OR discrimination OR discriminated OR disrespect* OR "disruptive behavior" OR unprofessional* OR hostil* OR cruel*) OR ((sex OR gender OR ethic* OR ethnic* OR obes* OR racial OR cultural) AND bias*)) NOT SU ((“domestic violence” OR (resident* AND “nursing home*”) OR “elder abuse”))NOT SU ((“internship” OR “intern” OR residents OR residency OR resident OR “medical student” OR “medical students” OR clerkship* OR trainee* OR housestaff OR “clinical fellow” OR fellows)) AND SU ((mistreat* OR cyberbully* OR bully* OR abuse OR maltreat* OR abused OR harass* OR humiliate* OR yelling OR intimidate* OR coercion OR racism OR discrimination OR

© 2018 Mazer LM et al. *JAMA Network Open.*

discriminated OR disrespect* OR "disruptive behavior" OR unprofessional* OR hostil* OR cruel*) OR ((sex OR gender OR ethic* OR obes* OR racial OR cultural) AND bias*)) NOT SU ((“domestic violence” OR (resident* AND “nursing home*”) OR “elder abuse”))

2. TI ((“internship” OR “intern” OR residents OR residency OR resident OR “medical student” OR “medical students” OR clerkship* OR trainee* OR housestaff OR “clinical fellow” OR fellows)) AND TI ((mistreat* OR cyberbully* OR bully* OR abuse OR maltreat* OR abused OR harass* OR humiliate* OR yelling OR intimidate* OR coercion OR racism OR discrimination OR discriminated OR disrespect* OR "disruptive behavior" OR unprofessional* OR hostil* OR cruel*) OR ((sex OR gender OR ethic* OR ethnic* OR obes* OR racial OR cultural) AND bias*)) NOT TI ((“domestic violence” OR (resident* AND “nursing home*”) OR “elder abuse”))

3. 1 OR 2

PSYCINFO

1. exp medical internship OR exp medical residency OR exp medical students OR (internship OR intern OR residents OR residency OR resident OR medical student OR medical students OR clerkship* OR trainee* OR housestaff OR clinical fellow OR fellows).ti
2. ((sex or gender or ethic* or ethnic* or obes* or racial or cultural) and bias*).ti
3. (exp bullying OR exp harassment OR exp relational aggression OR abuse of power/ OR teasing/ OR coercion/ OR oppression/ OR Victimization/ OR antisocial behavior/ OR abuse.hw OR discrimination.hw) OR (mistreat* or cyberbully* or bully* or abuse or maltreat* or abused or harass* or humiliate* or yelling or intimidate* or coercion or racism or discrimination or discriminated or disrespect* or "disruptive behavior" or unprofessional* or hostil* or cruel*).ti.
4. ("domestic violence" or (resident* and "nursing home") or "elder abuse").ti. OR (domestic violence/ OR elder abuse/ OR partner abuse/)
5. 1 AND (2 OR 3) NOT 4

I used the OVID limits to limit the search to English, Journal Article, Chapter, Dissertation, Reviews

COCHRANE LIBRARY

· Because Cochrane is so small relative to PubMed, SCOPUS, etc., I searched titles/abstracts/keywords

1. (internship OR intern OR residents OR residency OR resident OR “medical student” OR clerkship* OR trainee* OR housestaff OR “clinical fellow” OR fellows)

2. (mistreat* OR cyberbully* OR bully* OR abuse OR maltreat* OR abused OR harass* OR humiliate* OR yelling OR intimidate* OR coercion OR racism OR discrimination OR discriminated OR disrespect* OR "disruptive behavior" OR unprofessional* OR hostil* OR cruel* OR ((sex OR gender OR ethic* OR ethnic* OR obes* OR racial OR cultural) AND bias*))

© 2018 Mazer LM et al. *JAMA Network Open.*

3. (“domestic violence” OR (resident* AND “nursing home*”) OR “elder abuse”)

(internship OR intern OR residents OR residency OR resident OR “medical student” OR clerkship* OR trainee* OR housestaff OR “clinical fellow” OR fellows) AND (mistreat* OR cyberbully* OR bully* OR abuse OR maltreat* OR abused OR harass* OR humiliate* OR yelling OR intimidate* OR coercion OR racism OR discrimination OR discriminated OR disrespect* OR "disruptive behavior" OR unprofessional* OR hostil* OR cruel* OR ((sex OR gender OR ethic* OR ethnic* OR obes* OR racial OR cultural) AND bias*)) NOT (“domestic violence” OR (resident* AND “nursing home*”) OR “elder abuse”) NOT (internship OR intern OR residents OR residency OR resident OR “medical student” OR clerkship* OR trainee* OR housestaff OR “clinical fellow” OR fellows) AND (mistreat* OR cyberbully* OR bully* OR abuse OR maltreat* OR abused OR harass* OR humiliate* OR yelling OR intimidate* OR coercion OR racism OR discrimination OR discriminated OR disrespect* OR "disruptive behavior" OR unprofessional* OR hostil* OR cruel* OR ((sex OR gender OR ethic* OR obes* OR racial OR cultural) AND bias*)) NOT (“domestic violence” OR (resident* AND “nursing home*”) OR “elder abuse”)

1 AND 2 NOT 3

© 2018 Mazer LM et al. *JAMA Network Open.*
